# Dual-band dual-beam millimeter-wave antenna using dual circular microstrip rings

**DOI:** 10.1038/s41598-026-63512-0

**Published:** 2026-07-28

**Authors:** Amitkumar Patel, Chinthana Panagamuwa, William Whittow

**Affiliations:** Wolfson School of Mechanical, Electrical and Manufacturing Engineering, Loughborough University, Loughborough, LE11 3TU UK

**Keywords:** Engineering, Physics

## Abstract

The growing demand for high-capacity millimeter-wave (mmWave) communication systems requires compact, high-gain antennas that provide efficient spatial coverage while remaining simple to fabricate and integrate. This article presents a novel dual-band, dual-beam microstrip antenna based on a dual circular-ring radiator. Unlike existing designs, the proposed antenna achieves wide dual-band, dual-beam operation without vias, parasitic elements, or metamaterial loading, resulting in a compact, low-profile architecture that is straightforward to fabricate using standard printed circuit board (PCB) technology. Excitation of higher-order resonant modes, combined with optimization of the ring geometry and ground plane, produces symmetric dual-beam radiation in the quasi-E plane across two wide operating bands of 24.2–30 GHz and 32.5–43.5 GHz. Measured results agree closely with simulations, validating the antenna’s wide impedance bandwidth and stable radiation characteristics. The antenna generates dual beams at ± 40$^{\circ }$ in the lower band and ± 24$^{\circ }$ in the upper band, achieves a peak realized gain of 9 dBi at 38 GHz, and maintains gains exceeding 6 dBi across both bands. These characteristics make the proposed antenna a practical and cost-effective solution for next-generation indoor mmWave communication systems.

## Introduction

The swift advancement of millimeter-wave (mmWave) research has predominantly been motivated by the imperative to achieve higher data speeds and bandwidth essential for fifth generation (5G) and future mmWave communications^1,2^. Due to limited bandwidth availability and congested spectrum, sub-6 GHz RF communications fail to meet the demands of high data rates and massive wireless communication connectivity^3,4^. The enormous bandwidth available in the mmWave spectrum makes it a compelling option for enhancing data rates and increasing the capacity of 5G networks.

However, mmWave communication link quality is often negatively affected by intrinsic effects, such as increased free-space path loss, atmospheric absorption, and higher sensitivity to link blockage^5,6^. Although single-beam high gain antennas can alleviate free space path-loss, such links are particularly vulnerable to obstructions. A typical solution for improving channel capacity and link robustness is to use a MIMO system, which makes use of spatial diversity^7–9^. In^9^, the authors showed multi-beam antennas in MIMO systems can perform better at mmWave frequencies compared to high-gain single-beam and omnidirectional antennas. In^10^, the authors implement two orthogonal dual-beam antennas and show how they could be used in a MIMO systems. Dual-beam antennas increase gain, compensating for free space path-loss, while simultaneously increasing special diversity in MIMO systems.

**Fig. 1:**
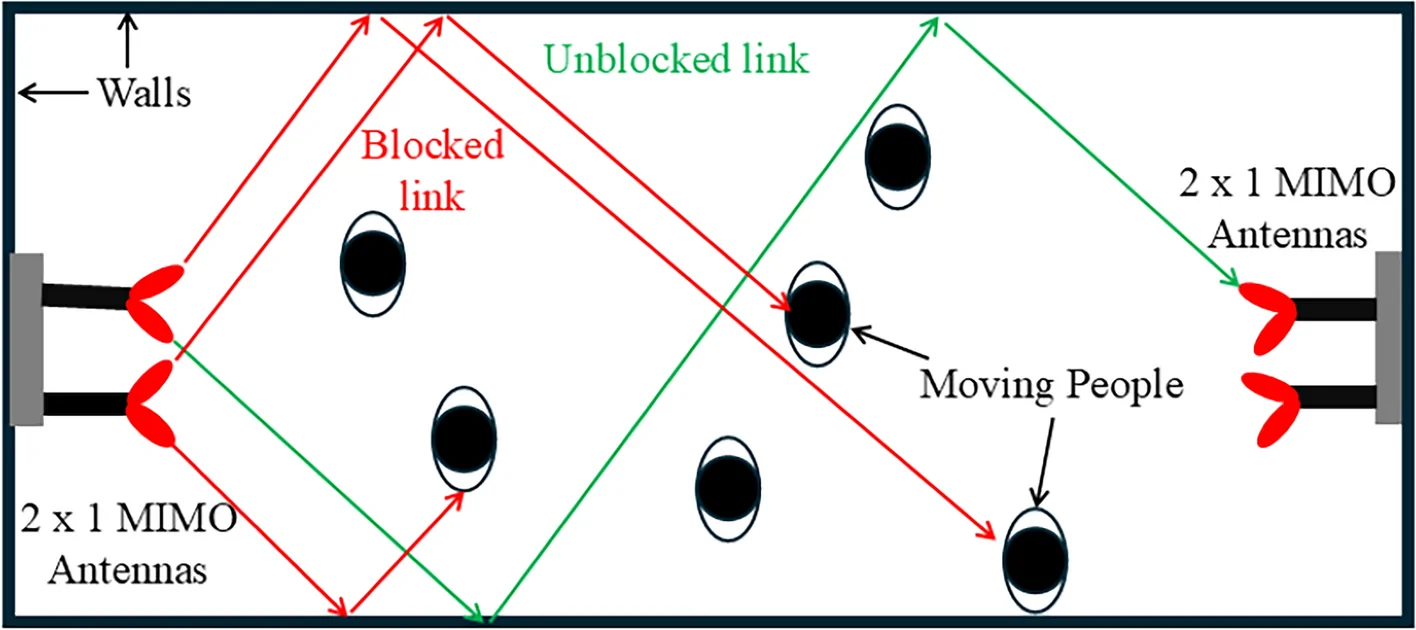
Potential application of a dual beam antenna to provide 5G NR connectivity to the podium area of a lecture theater.

Figure 1 shows the potential application of dual-beam antennas-based MIMO system for indoor wireless communications. Multiple directional beams compensate for transient obstructions through spatial diversity. Techniques for beamforming networks, phase shifters, and metamaterial (MTM) elements for generating dual-beam or multi-beam radiation have been presented^11–13^. However, they can suffer from complex design networks and higher losses^13^.

Microstrip patch antennas operating in the TM$_{02}$ or TM$_{20}$ modes have been realized for generating symmetrical dual-beam radiation^11,12^. However, they suffer from a narrow operating frequency range. To enhance the bandwidth of dual-beam mode operation, U-shaped slots or metallic strips have been incorporated^11–14^. The U-shaped slots exploit the TM$_{20}$ mode for achieving dual-beam radiation, however, asymmetry in the design leads to differences in the peak gains (by *∼* 2 dB) and radiation directions (2$^{\circ }$) of the two beams^10^. Symmetrical dual-beam radiation has also been attained using multi-layered structures, dipoles/bow-ties loaded with metamaterials, array and dielectric resonator loaded in patch antennas^14–22^. In^18^, authors have proposed a multilayered dual beam patch antenna which radiates in boresight directions. It achieved a peak gain of 9.7 dBi and up to 55.7% bandwidth. To radiate in the endfire direction, dual-beam endfire dielectric resonator antennas are proposed in^19–22^ that achieve high radiation efficiency and low loss in the mmWave band, but face the expense of complexity in the design.

Simple planar antennas based on thin substrates have been proposed to generate dual-beam radiation with wide bandwidths^23,24^. They exhibit stable and symmetrical radiation patterns over the operating frequency range. However, these antenna have lower gain. To improve gain performance, arrays of the same geometry have been created, achieving peak gain of more than 10 dBi^23,24^. However, they increase the overall size of the antenna.

In this paper, we present a simple and low-profile planar antenna, which exhibits dual-beam radiation in dual-bands. The main contributions of this proposed design are as follows:

1. The proposed antenna provides dual-band dual-beam operation in the mmWave frequency range using a simple single-layer, via-free planar structure, whereas many reported designs operate at a single band or require more complex multilayer or array configurations.
2. It achieves symmetrical dual-beam radiation with beam directions of approximately ± 40$^{\circ }$ at 28 GHz and ± 24$^{\circ }$ at 38 GHz, while maintaining low side-lobe levels and stable radiation characteristics over both operating bands.
3. It achieves symmetrical dual-beam radiation with minimal gain variation between the two beams. It has deep null at center that makes the proposed antenna useful for traditional mono-pulse radar for object detection.
4. The optimized dual circular-ring radiator together with the modified ground plane enhances both the impedance bandwidth and realized gain without requiring parasitic elements, metamaterials, electromagnetic band-gap structures, or multilayer substrates, thereby reducing fabrication complexity and cost. Wide-bandwidth over dual-bands and better gain performance keep the proposed antenna suitable choice in 5G/6G communication, vehicle to infrastructures (V2X) communication, and automotive radar applications.

The current limitations of the proposed antenna are summarized as follows:

1. The maximum realized gain is approximately 9.1 dBi, which can limit the use of the proposed antenna for some long-range mmWave communication systems. Higher gain could be achieved in future work through array implementation or the incorporation of metamaterial-based superstrates, albeit at the expense of increased size and complexity.
2. The proposed antenna provides fixed dual-beam radiation. Future work may investigate beam reconfigurability by incorporating PIN or varactor diodes into the circular-ring structure, enabling dynamic control of the beam direction or the central null for applications such as beam-steering and object tracking.

This paper is organized as follows. The section, Antenna Mechanism for Dual-beam Configuration, describes the design methodology and operating mechanism of the proposed dual-beam antenna. The section, Fabrication and Experimental Validation of the Dual Circular-ring Antenna, presents the fabrication of the prototype and its experimental characterization. Simulated and measured results are compared in this section to validate the proposed design. The section, Comparison and Discussion, compares the proposed antenna with previously reported designs and discusses its performance. Finally, the section, Conclusion, summarizes the main findings and conclusions of this work.

## Antenna mechanism for dual-beam configuration

This section gives an overview of the proposed antenna and its operating principles.

The design strategy of the proposed microstrip ring antenna is based on the theoretical framework carried-out in^25–28^. When the substrate thickness of the antenna is very small compared to the operating wavelength and the outer peripherals act as magnetic walls, the circular cavity model can be used to study the operating modes of the ring antenna. These works described the methods for generating and elucidating the radiation patterns of various higher-order modes. A circular-ring with a finite ground plane and a thin substrate exhibits no field variation in the z-direction (perpendicular to the geometry), thereby supporting TM$_{nm}$ modes. Here, the integer n and m denote the field variation in the azimuthal and radial directions, respectively.

**Fig. 2:**
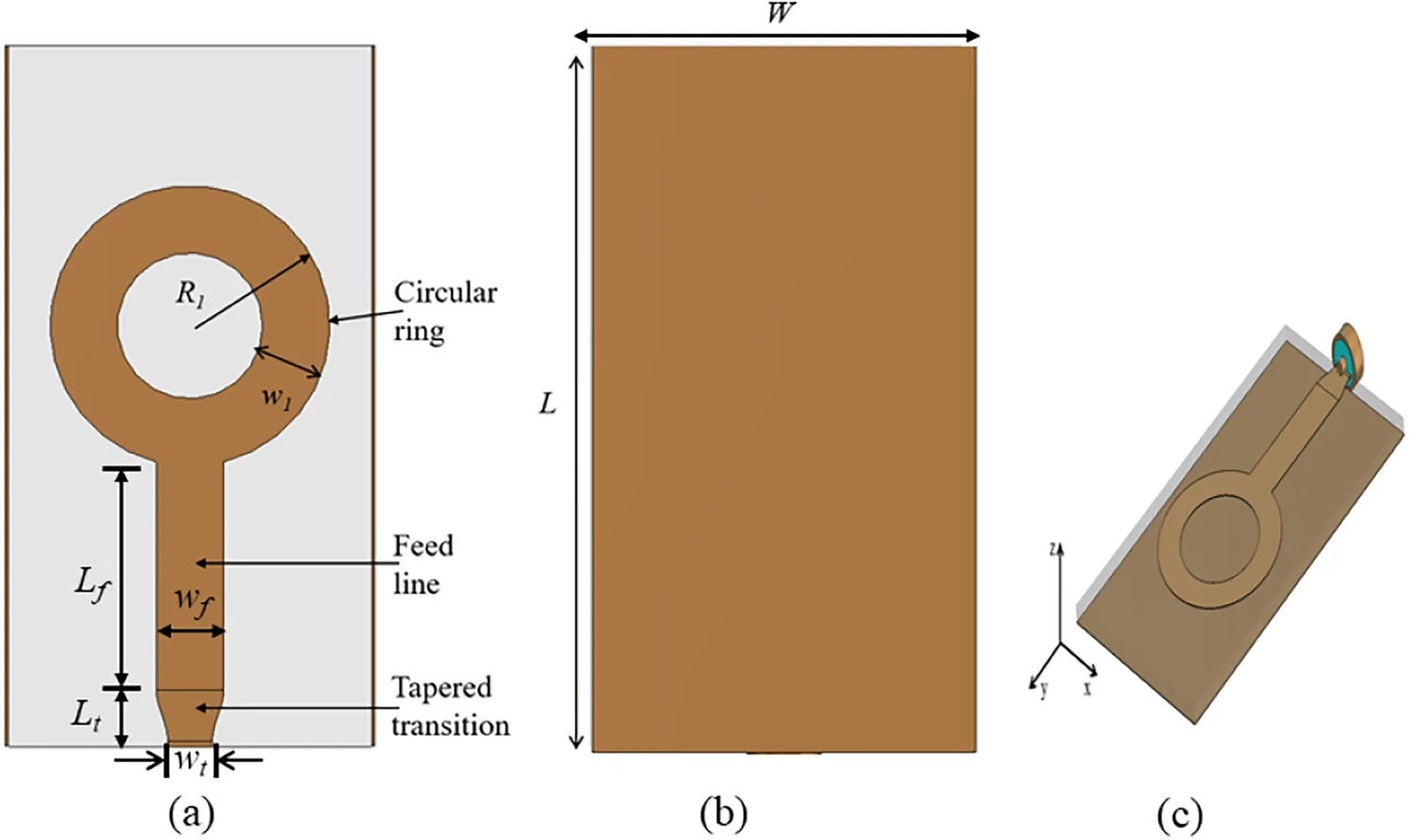
Geometry of the circular-ring patch antenna (**a**) Top-view; (**b**) Bottom view; and (**c**) Perspective view with RF connector.

The TM$_{11}$ mode emerges as the dominant mode for the circular-ring which is characterized by its minimum radius. The ring antenna operated in the TM$_{11}$ mode offers a high input impedance and narrow bandwidth. The inner and outer radii of the ring define its resonant behavior and separation of resonant modes. Moreover, its performance can be tailored by adjusting geometric parameters, feed configuration, and substrate properties. To generate dual or multiple beams in the elevation plane, the structure must support higher-order modes. The crucial aspect of circular-ring-based planar antennas lies in the radius of the ring, which dictates the flow of higher-order mode currents. When this antenna operates in the TM$_{21}$ mode, it provides several times higher bandwidth compared to TM$_{11}$ mode but with a cost of larger size. However, it has been shown that this antenna is a good resonator, thus storing energy, for TM$_{1m}$ modes (m odd) and a good radiator for TM$_{1m}$ modes (m-even)^26^. The radius of a circular-ring is determined by (1)^26^.

1 $$\begin{aligned} R_{i} = \frac{\chi _{nm}c}{2\pi f_{nm} \sqrt{\varepsilon _{r}}} \end{aligned}$$

where *c* is the velocity of light in free space, $\varepsilon _{r}$ the relative permittivity of the substrate, $\chi _{nm}$ is the m$^{th}$ zero of the derivative of nth order of the Bessel’s function. The surface current flow for various higher-order modes such as TM$_{21}$, TM$_{13}$, TM$_{12}$, TM$_{02}$ and other modes was described in^26^. Higher order modes like TM$_{21}$, TM$_{02}$ and TM$_{41}$ have the capability to produce dual-beam or conical-beam radiation in the elevation-plane^26^. Understanding these current flows helps optimize the design of the circular-ring antenna to radiate in the higher-order modes. The single circular microstrip ring with a full ground plane is shown in Fig. 2.

**Fig. 3:**
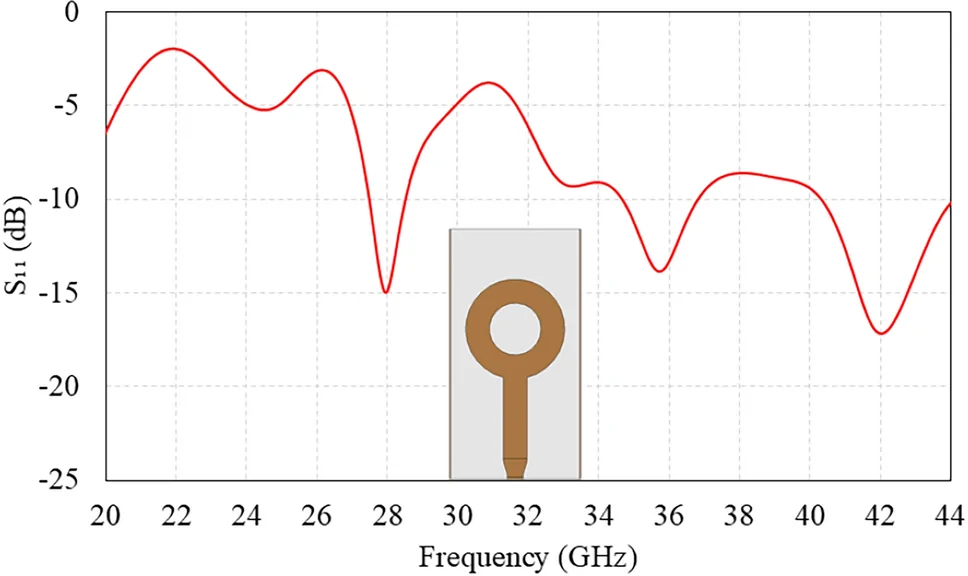
The simulated reflection coefficient of the circular-ring antenna having microstrip feedline and tapered transition.

**Fig. 4:**
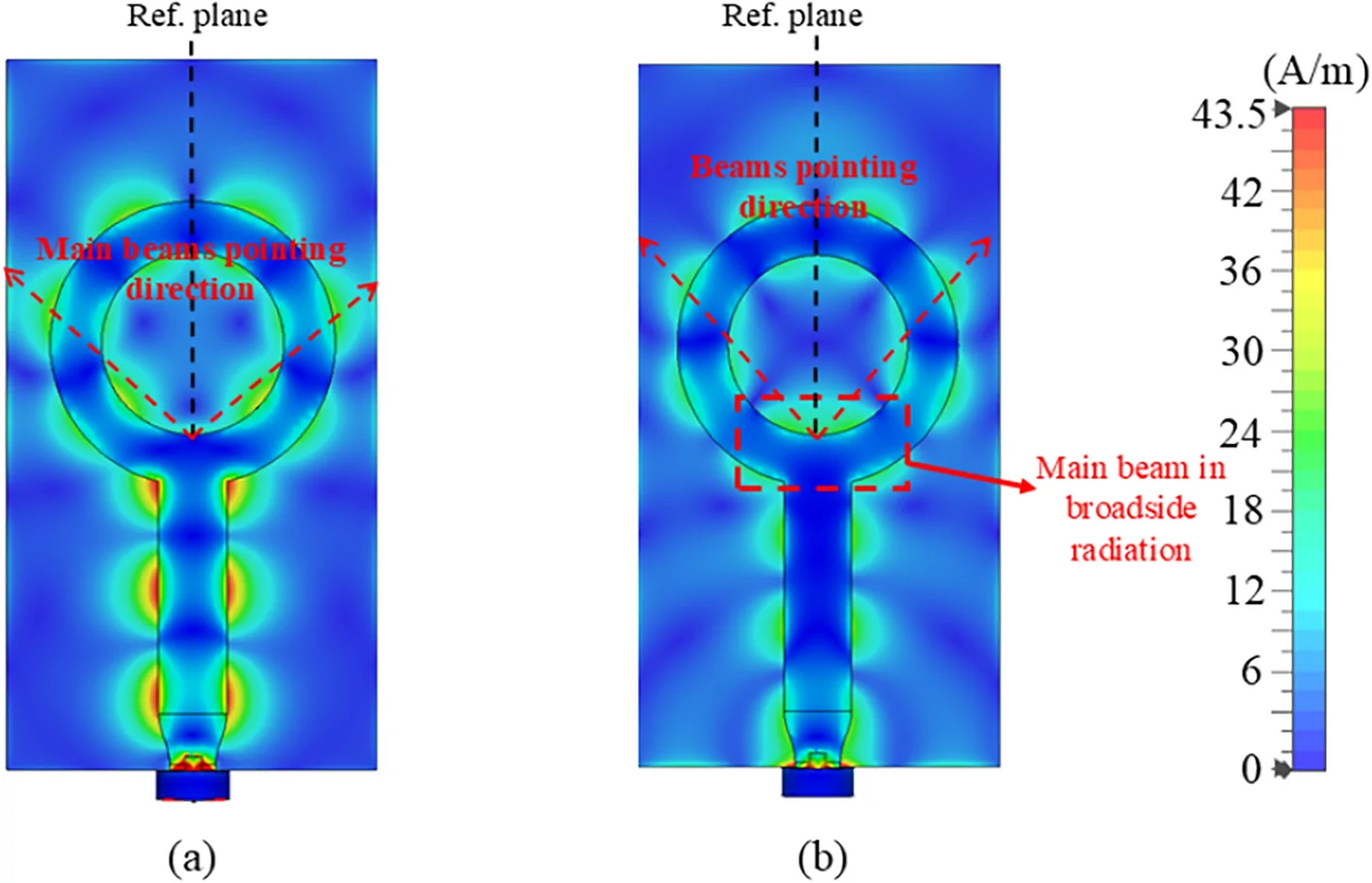
The simulated currents distribution on the surface of the circular-ring antenna at (**a**) 28 GHz; and (**b**) 36 GHz.

The top-view illustration in Fig. 2a consists of the circular-ring whose radius is derived based on  (1), a 50 *Ω* microstrip feed line and a linear-tapered transition. The design was implemented on Rogers RT Duroid 5880 substrate with a thickness of 0.787 mm and a dielectric constant of 2.2. The antenna is designed and evaluated in Computer Simulation Technology (CST) Microwave Studio software. The reflection coefficient of the antenna is shown in Fig. 3. It gives a -10 dB impedance bandwidth (IBW) from 27.5 to 28.5 GHz, 34.7 to 36.9 GHz and 40.2 to 44 GHz. Figure 3 shows the antenna has multiple resonance peaks due to excitation of the fundamental and higher order modes. Figure 4 illustrates the azimuthal surface current density concentrated along the periphery of the circular-ring at 28 GHz and 36 GHz, demonstrating that the single circular-ring patch antenna strongly supports higher order modes (TM$_{1m}$ where m is even).

**Fig. 5:**
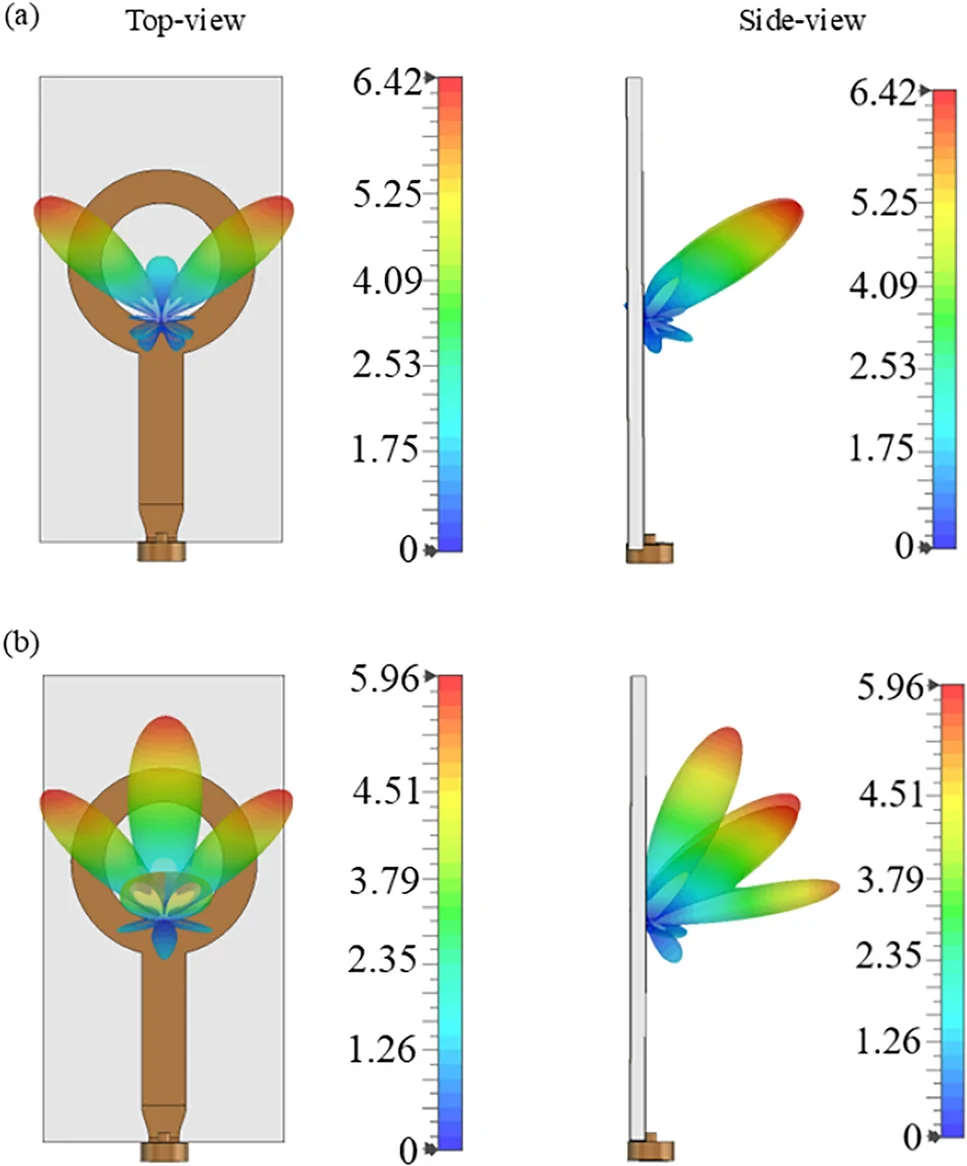
Simulated 3D linear gain radiation patterns of the circular-ring antenna at (**a**) 28 GHz; and (**b**) 36 GHz.

The 3D linear gain radiation patterns are presented in Fig. 5. Figure 5a shows the antenna has dual-beams at 28 GHz but is tilted towards boresight (*θ* = 30$^{\circ }$ in the elevation plane). Furthermore, at 36 GHz, an unwanted additional beam is produced at *ϕ* = 90$^{\circ }$ in the azimuth plane and it also radiates at *θ* = 30$^{\circ }$ in the elevation plane (for better visualization of radiation pattern, the 3D gain plot is shown in linear scale).

**Fig. 6:**
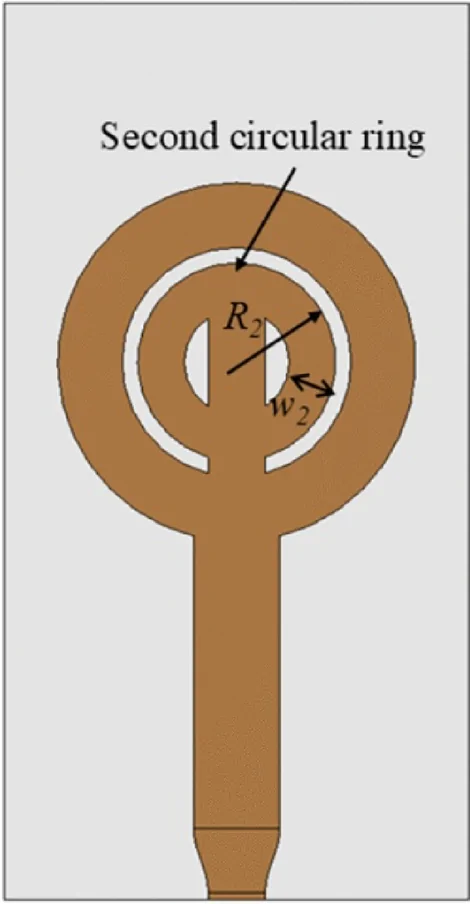
Geometry of the dual circular-ring antenna fed with a microstrip line.

**Fig. 7:**
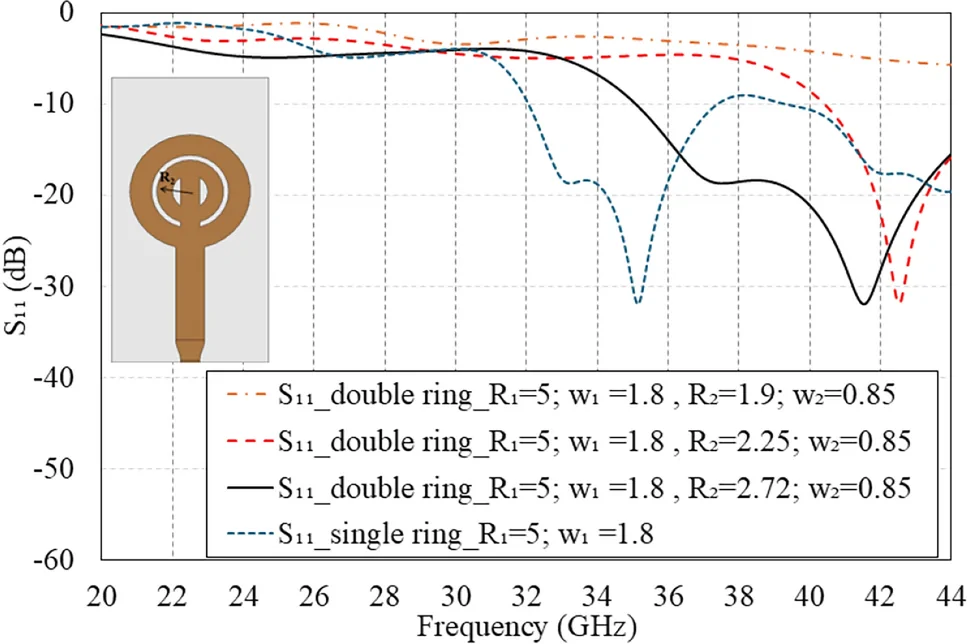
Simulated reflection coefficients with respect to variation in the second circular-ring ($R_{2}$).

To remove the additional beam, a second circular-ring has been incorporated as shown in Fig. 6. The impact of the second circular-ring’s radius variation on the reflection coefficient is shown in Fig. 7. It gives better IBW when the second ring radius is 2.72 mm with a thickness of 0.85 mm. However, it no longer radiates at 28 GHz and the radiation beams are still oriented towards boresight.

From the above investigations, it is clear that the proposed antenna operates through the combined action of the dual circular-ring radiator and the modified ground plane. The larger outer ring establishes the dominant resonance around 28 GHz, while the addition of the inner circular-ring introduces a second resonant current path that excites a higher-order circumferential mode near 38 GHz. At both operating frequencies, the surface currents circulate around the rings with approximately symmetric phase progression, producing two dominant radiation regions. The constructive interference between these radiating regions results in two tilted main beams rather than a conventional broadside radiation pattern.

**Fig. 8:**
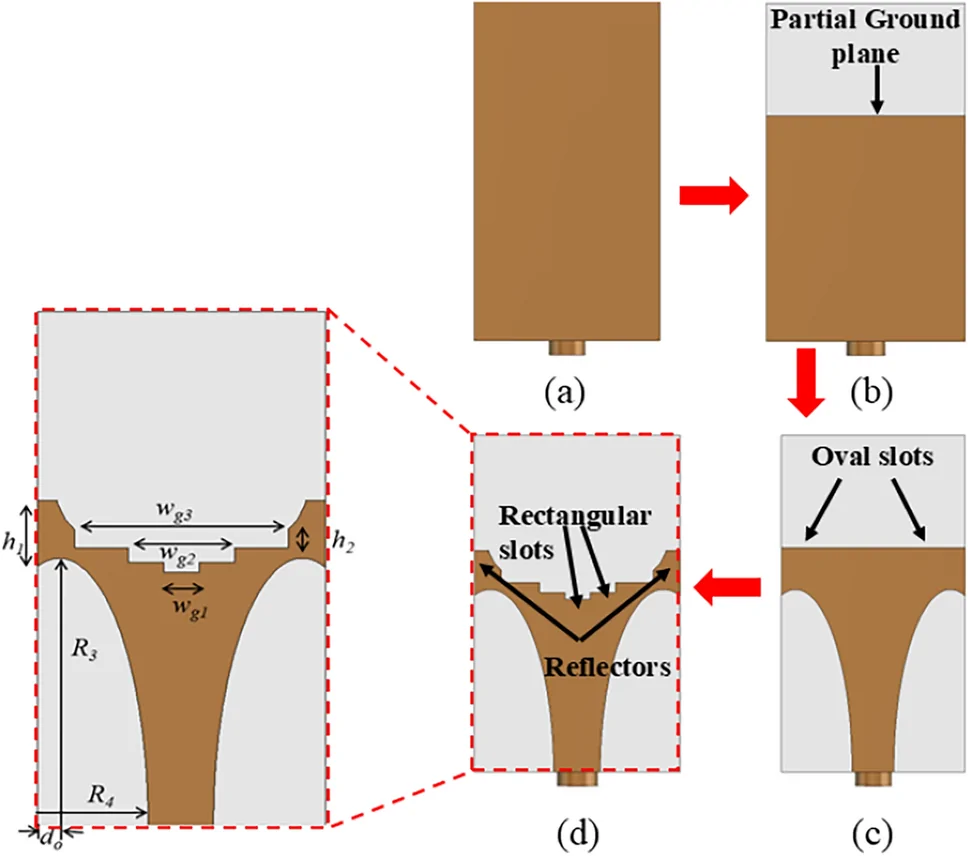
Evolution of ground plane in terms of slots and reflectors.

**Fig. 9:**
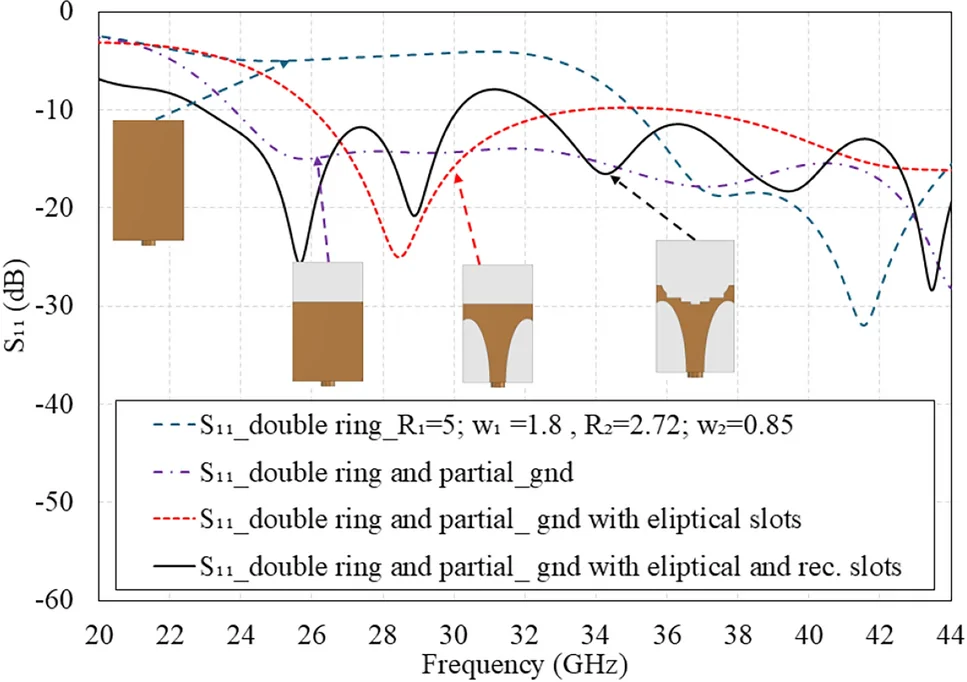
Simulated reflection coefficients with respect to variation in ground plane of the dual circular-ring antenna.

**Fig. 10:**
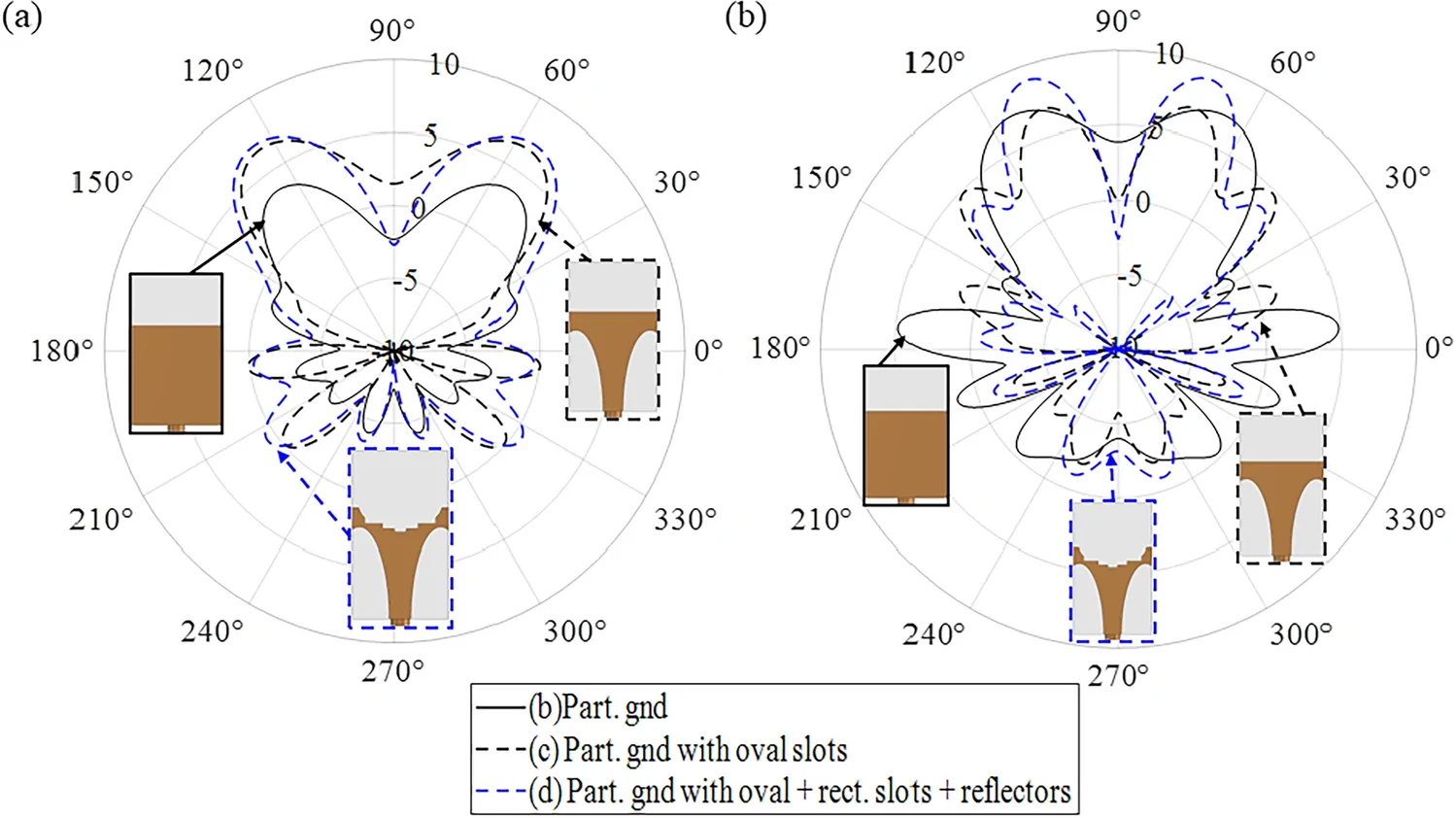
Simulated 2D radiation patterns with respect to variation in ground planes (as per Fig. 8) of the dual circular-ring antenna (**a**) 28 GHz; and (**b**) 38 GHz.

To steer the dual beams towards the endfire direction (*θ* = 90$^{\circ }$), the ground plane plays an equally important role in controlling the radiation characteristics. Hence, two oval and two rectangular slots and reflectors were introduced to the ground plane, as shown in Fig. 8. The evolution from a full ground plane to a partial ground plane initially shifts the radiation toward the endfire direction by modifying the return-current path. Fig. 9 illustrates the reflection coefficients of the antenna as the ground plane evolves. As illustrated in Fig. 9, Subsequently, the introduction of the oval slots improves impedance matching and redistributes the ground currents, whereas the rectangular slots together with the reflector sections introduce additional phase control beneath the radiator. Notably, the final design yields a −10 dB IBW ranging from 24.2 to 30.2 GHz, and 31.4 to 44 GHz. Figure 10 represents the 2D radiation patterns of the antenna at each evolving stage of ground plane at 28 GHz and 38 GHz. From Fig. 10, it is clear that the progressive modification suppresses the undesired broadside lobe while reinforcing radiation in the two off-axis directions. Consequently, the beam direction gradually moves from approximately 15$^{\circ }$ to 80$^{\circ }$ - 85$^{\circ }$, the gain increases from 6 dBi to 9.2 dBi, and the side-lobe levels improve from approximately −3 dB to −10.8 dB, as summarized in Table 1.

**Fig. 11:**
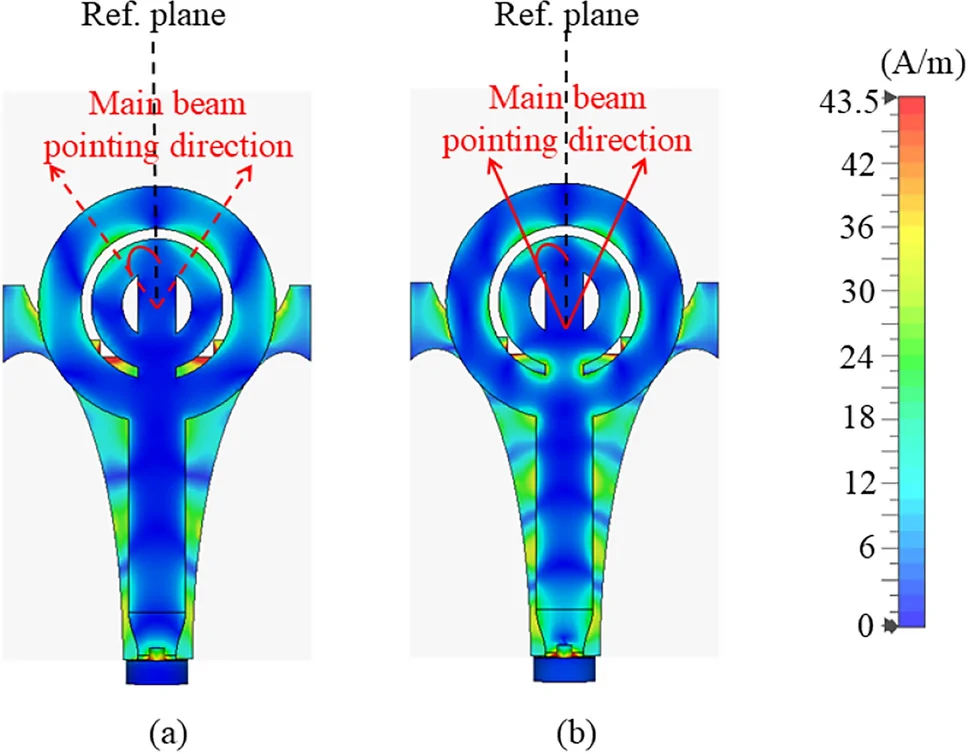
The simulated current distributions on the surface of dual circular-ring antenna at (**a**) 28 GHz; and (**b**) 38 GHz.

**Fig. 12:**
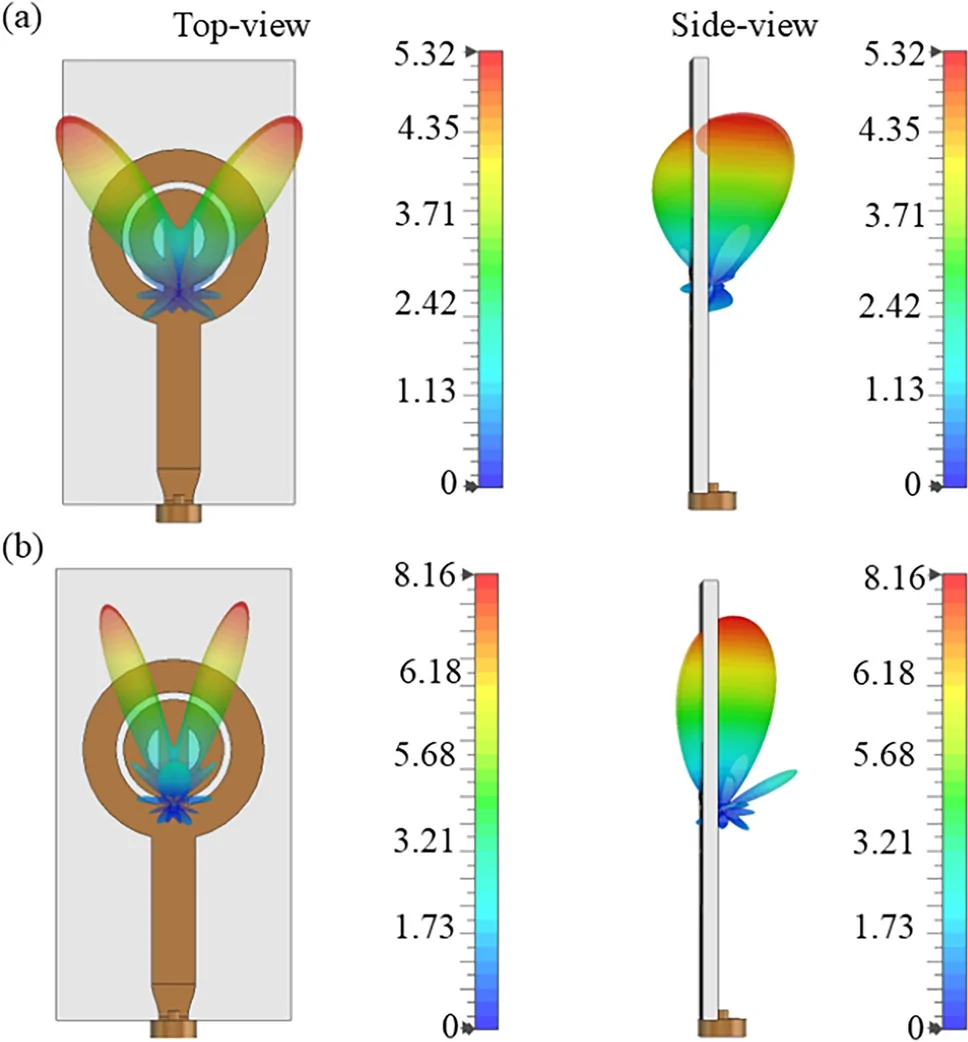
Simulated 3D linear realized gain radiation patterns of the dual circular-ring antenna at (**a**) 28 GHz; and (**b**) 38 GHz; 44 GHz.

**Fig. 13:**
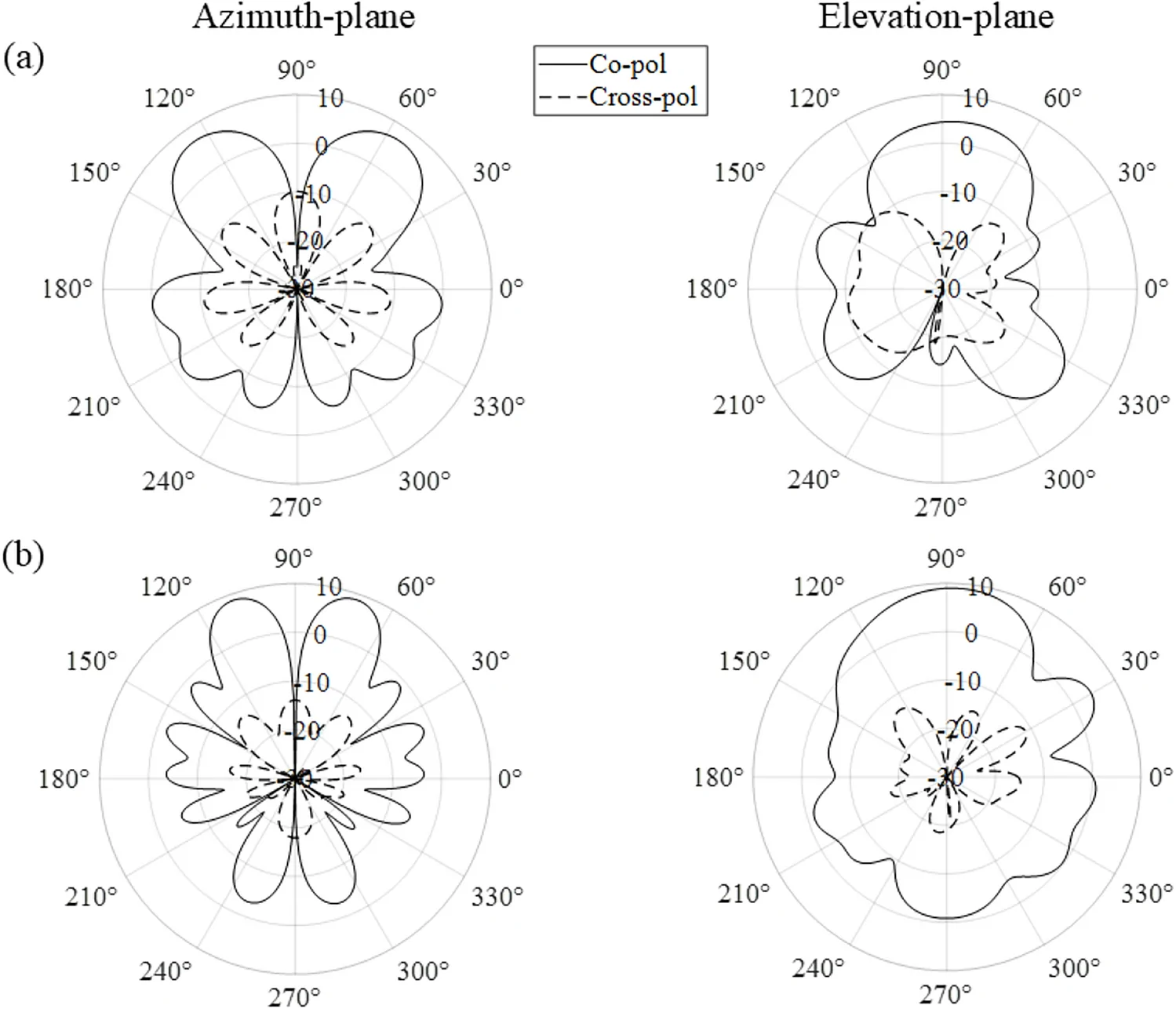
Simulated 2D radiation patterns of the dual circular-ring antenna at (**a**) 28 GHz; and (**b**) 38 GHz.

The surface current distributions on the proposed dual circular-ring at 28 GHz and 38 GHz are shown in Fig. 11. Figure 11b confirms that the optimised second circular-ring and ground plane in the antenna geometry eliminates the current hot spot at the junction of the feedline and the first circular-ring, previously responsible for the unintended boresight radiation. The refined configuration ensures a more symmetric current distribution at both frequencies, which supports stable dual-beam endfire radiation. Notably, these modifications preserve the antenna’s performance in the lower frequency band. The 3D linear gain plots at 28 GHz and 38 GHz, depicted in Fig. 12. Although the proposed antenna is well matched beyond 44 GHz, it cannot maintain the dual-beam radiation in endfire direction after 43.5 GHz and raises the level of side-lobes significantly. Hence, we have kept the results up to 44 GHz. Figure 13 shows the simulated radiation patterns of co-pol and cross-pol components in both the planes at 28 GHz and 38 GHz. Compared to the gain in the main beam direction, the cross-pol components are always *<-*15 dB in the lower frequency band and *<-*20 dB in the upper frequency band, thus demonstrating good polarization purity.

**Table 1:** Impact of ground-plane design on the proposed antenna.

Ground plane	Frequency (GHz)	−10 dB IBW	Beam direction ($\theta , \phi$) ($^{\circ }$)	Gain (dBi)	Side-lobe level (dB)
Partial (b)	28	*23.8 - 44*	(*55, ± 50*)	4.2	*-3.5*
	38		(*63, ± 30*)	7.2	*-5.5*
Partial + Oval slots (c)	28	*26 - 34*	(*80, ± 45*)	5.5	*-4.2*
	38	*36.2 - 44*	(*77, ± 30*)	5.8	*-6.6*
Partial + Oval + Rectangular slots + Reflectors (d)	28	*23 - 30*	(*80, ± 40*)	7.2	*-7.5*
	38	*32.8 - 44*	(*85, ± 24*)	9.2	*-10.8*

(b), (c) and (d) are as per Fig. 8

### Dual circular-ring antenna configuration

**Fig. 14:**
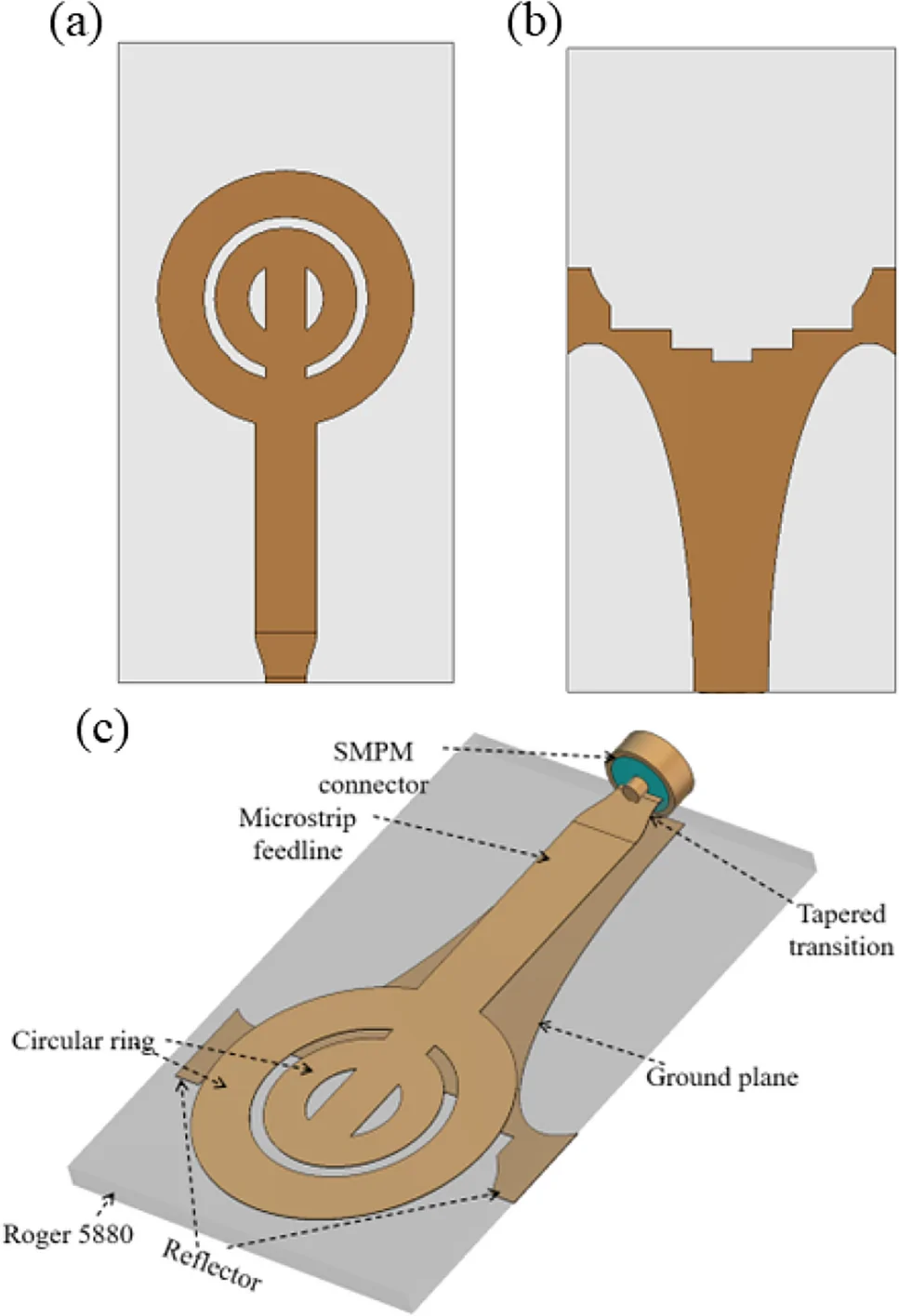
The final geometry of the dual circular-ring antenna (**a**) Top-view; and (**b**) Bottom view; and (**c**) Exploded view.

The final configuration of the dual circular-ring antenna is illustrated in Fig. 14. The overall size of the structure is 25 *×* 12 *×* 0.787 mm$^{3}$ and additional internal dimensions of the antenna are given in Table 2.

**Fig. 15:**
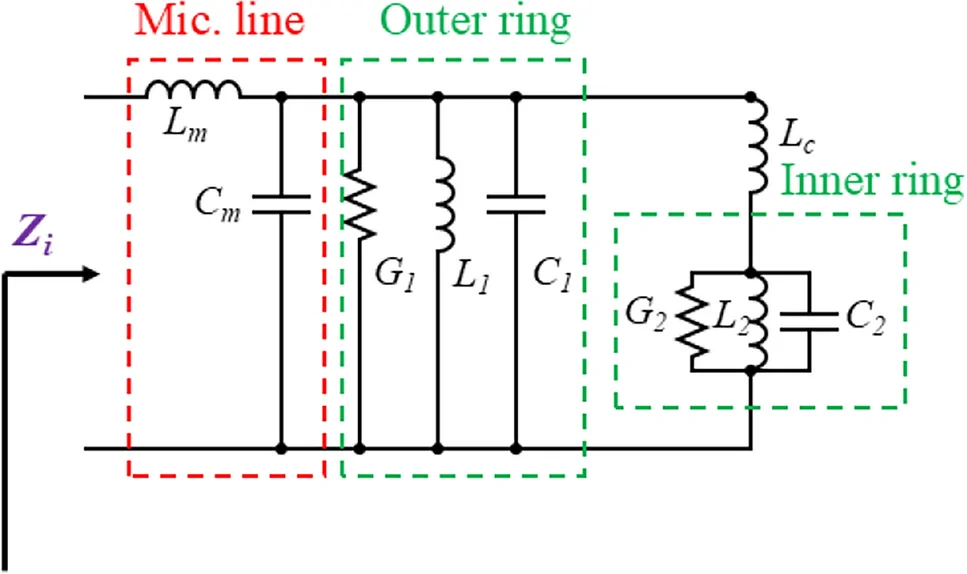
Lumped equivalent input impedance model of the dual circular-ring antenna.

The 50 *Ω* microstrip feed line is 5 mm long and 1.4 mm wide. A small linear-tapered transition is designed to facilitate the connection between the RF feed connector and microstrip line. An equivalent input impedance model of the proposed antenna is derived based on^18^, as shown in Fig. 15. The microstrip feed line is represented by a series inductance ($L_{m}$) and a shunt capacitance ($C_{m}$). Each circular-ring is modeled by a parallel combination of conductance (*G*), inductance ($L \approx \mu _0 R_{i} \left( \ln \left( \frac{8R_{i}}{w_{i}}\right) - 2 \right)$, where *i* =1, 2), and capacitance ($C \approx \frac{2\pi \varepsilon _0 \varepsilon _{\text {eff}} R_{i}}{\ln \left( \frac{8R_{i}}{w_{i}}\right) }$, where $\varepsilon _{\text {eff}} = \frac{\varepsilon _r + 1}{2}$). The physical coupling between the two rings is represented by an additional inductance ($L_c \approx 2 \times 10^{-7} \, l \left( \ln \left( \frac{2l}{w+t}\right) + 0.5 \right)$, where *t* is copper thickness = 0.035 *μ*m) between the two rings. Due to the complexity of accurately determining the exact values of the lumped elements, a generalized equivalent circuit model is presented.

**Fig. 16:**
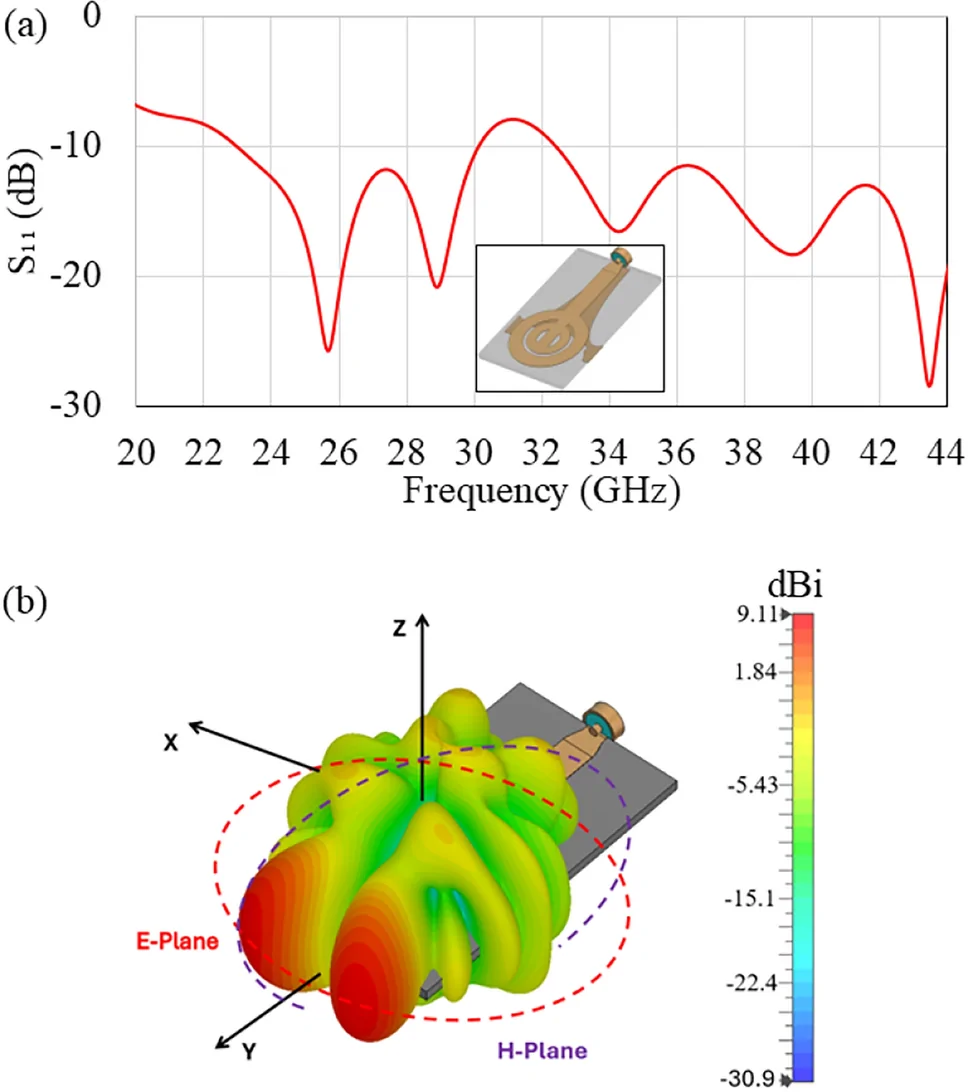
Simulated (**a**) reflection coefficient; and (**b**) 3D realized gain pattern at 38 GHz of the dual circular-ring antenna.

**Table 2:** Optimized Physical Dimensions of the Proposed Dual Circular-ring Antenna (See Figs. 2, 6 and 8 for dimension labels).

Parameter	Dimension (mm)	Parameter	Dimension (mm)	Parameter	Dimension (mm)
*L*	25	$R_{3}$	10	$h_{1}$	3.21
*W*	13	$R_{4}$	5	$h_{2}$	0.9
$R_{1}$	5	$L_{f}$	8.15	$w_{g1}$	1
$R_{2}$	2.72	$w_{f}$	2.4	$w_{g2}$	4.8
$w_{1}$	1.8	$L_{t}$	2	$w_{g3}$	9.6
$w_{2}$	0.85	$w_{t}$	1.4	$d_{0}$	1

The simulated reflection coefficient shown in Fig. 16a demonstrates that the proposed antenna achieves a − 10 dB IBW from 24.2 to 30.2 GHz and 31.4 to 44 GHz, covering the n258 (24.25–27.5 GHz), n257 (26.5–29.5 GHz), n261 (27.5–28.35 GHz), and n260 (37–40 GHz) bands of 5G NR mmWave communications.. Moreover, Fig. 16b shows the 3D realized gain pattern at 38 GHz which confirms the proposed antenna produces endfire dual-beams with very little back radiation (less than 15 dB.

## Fabrication and experimental validation of dual circular-ring antenna

**Fig. 17:**
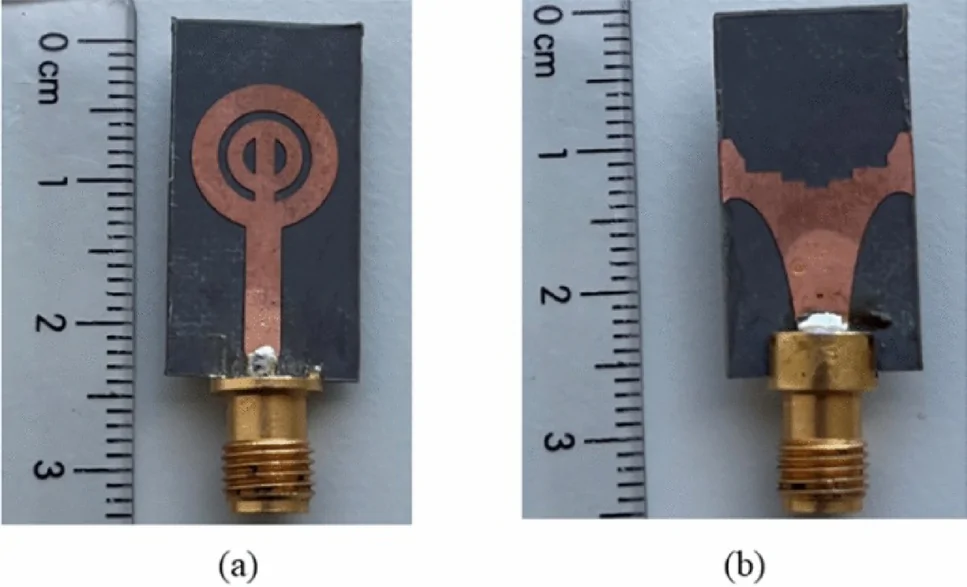
Fabricated prototype model of the dual circular-ring antenna.

**Fig. 18:**
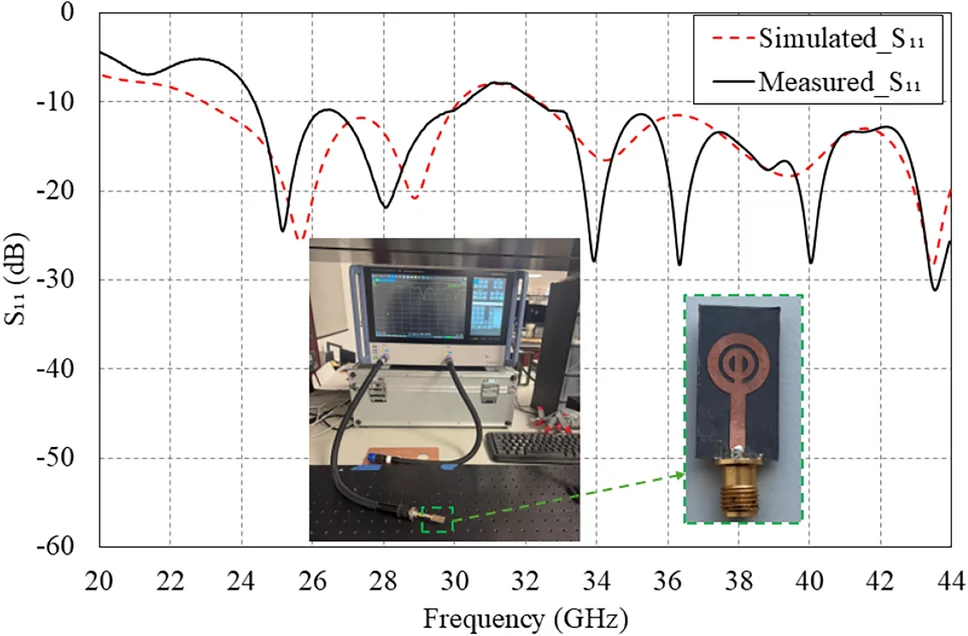
Comparison of simulated and measured reflection coefficients of a dual circular-ring antenna.

For demonstration purposes, a protype model of the proposed design was fabricated as shown in Fig. 17. The structure is realized on Rogers RT Duroid 5880 substrate having a dielectric constant of 2.2 and thickness of 0.787 mm. To excite the antenna, we have used a 1.85 mm connector that operates up to 65 GHz with an insertion loss better than 0.8 dB. The reflection coefficient of the proposed design has been measured using a ZNA vector network analyzer from Rohde & Schwarz. Figure 18 presents both the simulated and measured reflection coefficients.

The proposed antenna offers a wide −10 dB IBW for both bands, covering frequency ranges from 24.25 to 29.9 GHz (which covers the n258 and n257 bands) and 32.6 to 42.8 GHz (which covers the n260 and n261 bands). The simulated and measured results shown in Fig. 18 indicate a good match across the whole operating band.

The disagreement between the simulated and measured results are mainly attributed to practical fabrication and measurement uncertainties. At mmWave frequencies, small dimensional variations introduced during PCB fabrication, together with tolerances in the substrate dielectric constant and thickness, can slightly modify the effective electrical length of the antenna, leading to minor shifts in the resonant frequencies. In addition, the small male aperture (SMA) connector transition and manual soldering introduce unavoidable parasitic reactance, resulting in small variations in the measured reflection coefficient.

**Fig. 19:**
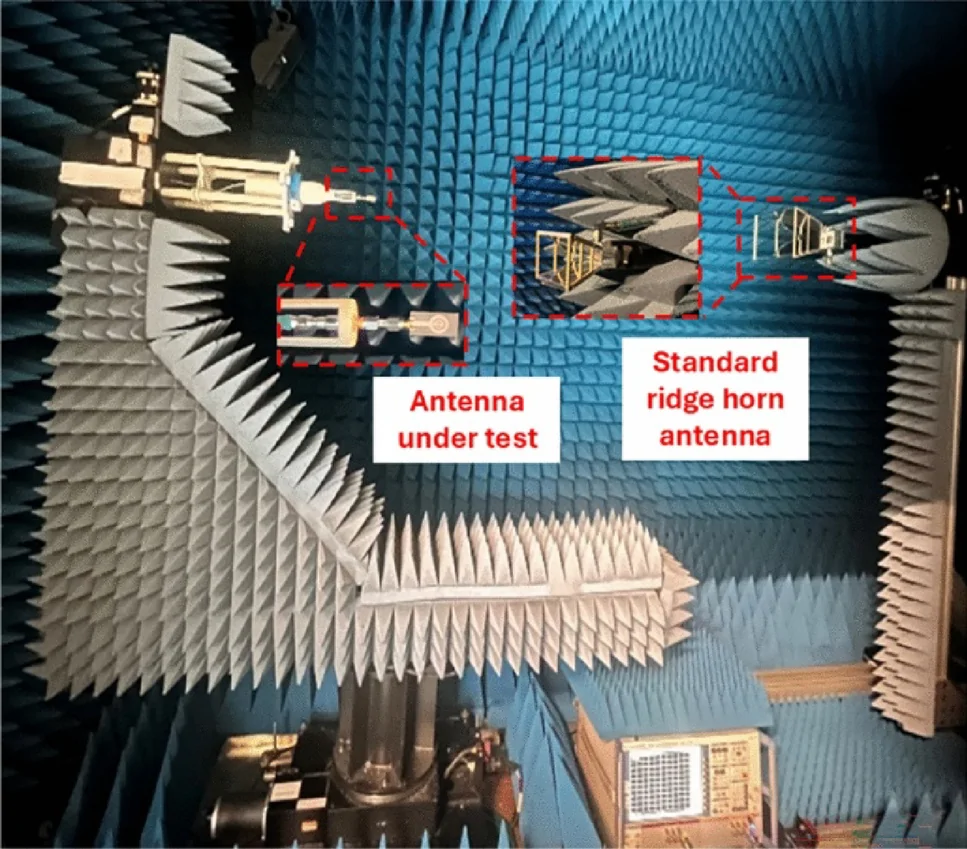
Test setup to carry out realized gain pattern measurements in both the principal planes of the proposed antenna in the anechoic chamber at Loughborough University.

**Fig. 20:**
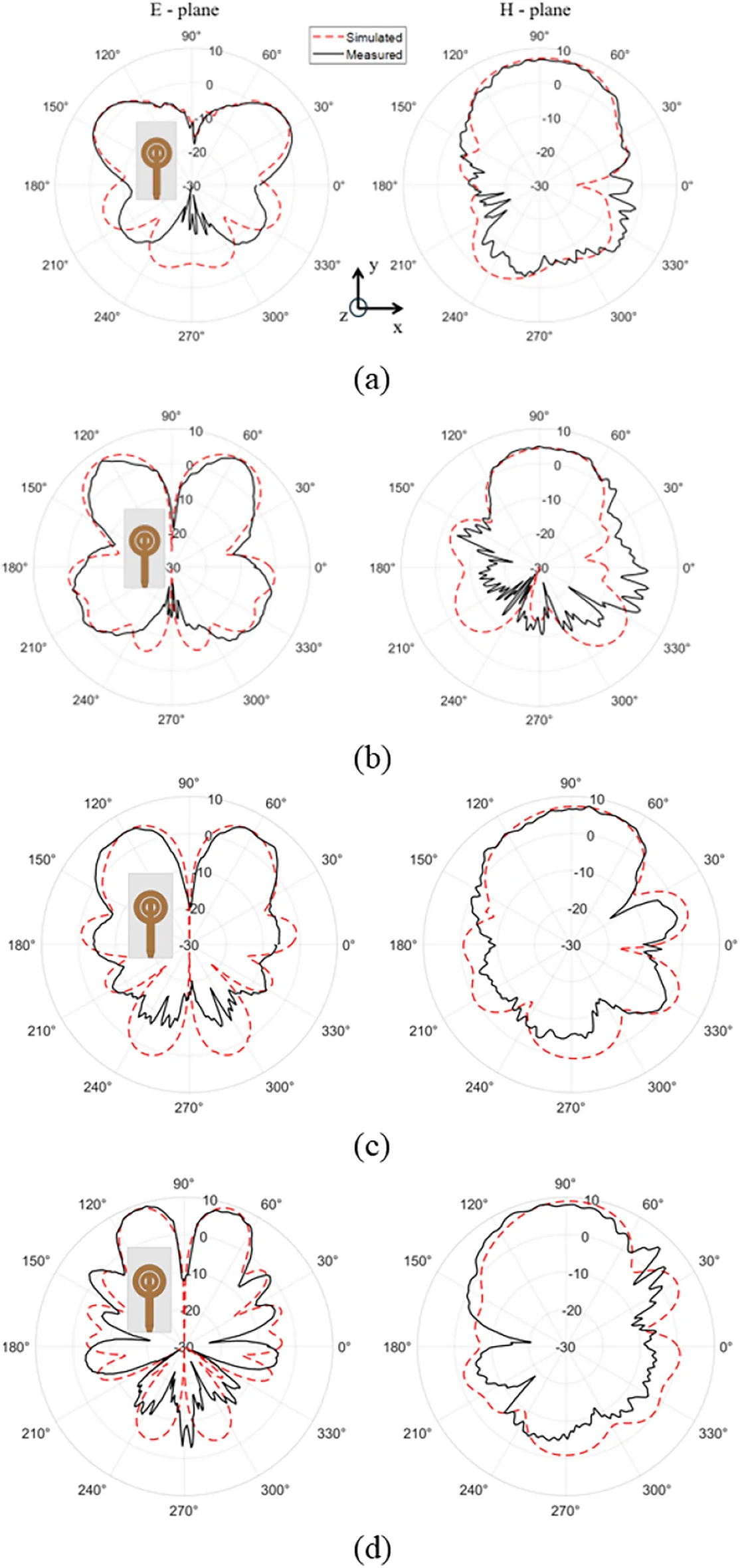
Simulated and measured radiation patterns of the dual circular-ring antenna at (**a**) 26 GHz; (**b**) 28 GHz; (**c**) 38 GHz; and (**d**) 40 GHz in the azimuth-plane and in the elevation-plane.

The radiation patterns and gain of the proposed antenna were measured using the anechoic chamber facility at Loughborough University. The measurement setup for the antenna is shown in Fig. 19. The simulated and measured azimuth plane radiation patterns of the proposed antenna at 24.5, 28, 32 and 38 GHz are presented in Fig. 20a–d, respectively.

It is clear that the simulated and measured patterns show close similarity in the direction of the main dual beams. Moreover, from the measured radiation patterns, it can be seen that the dual-beam radiation in both bands are radiated in the endfire direction, with the main lobe direction ± 40$^{\circ }$ in the lower band and ± 24$^{\circ }$ in the upper band.

**Fig. 21:**
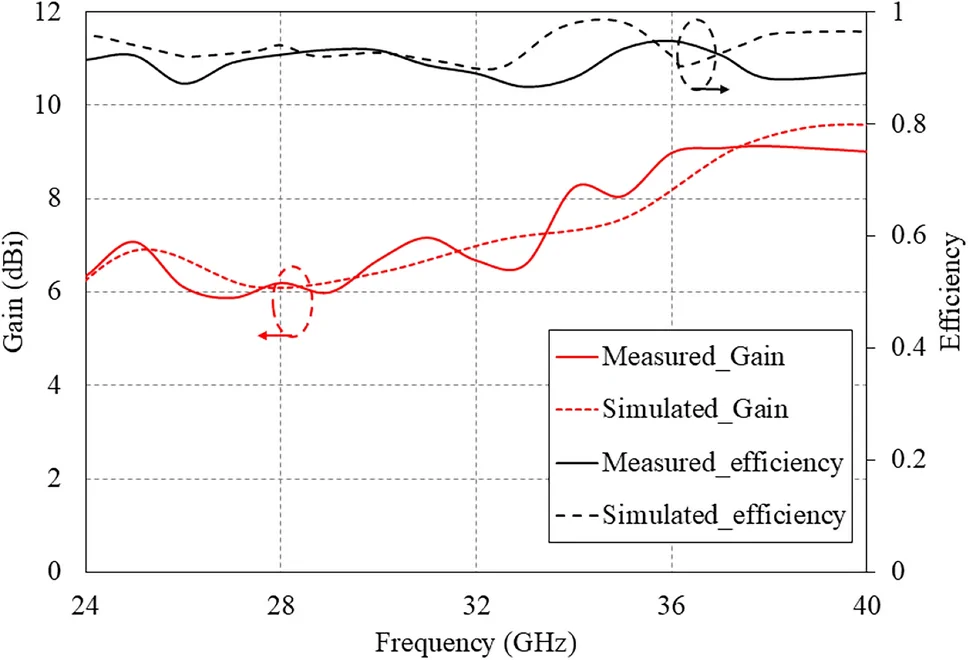
Simulated and measured gain and efficiency of the proposed antenna versus frequency plot.

During the radiation-pattern measurements, the antenna was mounted on a metallic fixture (in black as shown in Fig. 19) that holds the antenna in place as shown in Fig. 19. This is part of the chamber design and can not be altered. It is *∼* 10 times larger than the antenna and causes significant shielding and scattering when the test antenna is facing away from the standard ridge horn antenna. Although the fixture was carefully positioned to minimize its influence, residual scattering from the mounting structure and connector assembly cannot be completely eliminated and contributes to the slight differences observed in the measured back-lobe levels and realized gain.The minimum and maximum gains are achieved at 24.5 GHz and 38 GHz and are 6.5 dBi and 9 dBi, respectively. The largest cross-polar component is -15 dB down from the maximum co-pol gain direction at all frequencies hence, they are not shown for better clarity of plots. The proposed antenna gives a symmetrical dual-beam radiation pattern at all operating frequencies in the considered range. The gain and efficiency versus frequency curves are shown in Fig. 21. Overall, the measured results show good agreement with the simulated predictions, confirming the validity of the proposed antenna design within the expected experimental uncertainty.

## Comparison and discussion

The proposed antenna is tailored for dual-band dual-beam applications, featuring endfire radiation patterns. While existing literature supports several single or double beam antennas employing various design techniques and technologies, the proposed antenna stands out for its competitive performance. A comparison of the proposed antenna’s performance with other reported works is provided in Table 3, summarizing factors such as design technique, geometry size, single/dual beams in single/dual bands, bandwidth, peak gain and radiation efficiency.

**Table 3:** Performance Comparison of the Proposed Dual Circular-ring Antenna with Other Reported Works.

Pub.Year & Refs.	Design technique	Size of antenna ($\lambda _{0}^3$)	No. of layers	Bands	Beams / MBD ($^{\circ }$)	Bandwidth (GHz)/ FBW	Peak Gain (dBi)	Rad. Effi. (%)
2018^16^	Patch with slots	1.4 *×* 1.4 *×* 0.15	2	Single	Dual (± 47)	27.8 – 30.9 (10.6%)	8.8	N/A
2019^17^	Dipole & MTMs	2.6 *×* 2.6 *×* 0.04	1	Single	Dual (± 30)	24.3 – 27.5 (12.5%)	7.4	> 90
2020^23^	Fractal patch	2.98 *×* 1.12 *×* 0.023	1	Single	Dual (± 50)	25.3 – 29 (13.4%)	10.1	> 80
2020^29^	Patch & multi-layered (3D)	1.6 *×* 1.98 *×* 1.58	1	Single	Dual (± 15)	27.1 – 29.6 (8.6%)	11.6	90
2021^30^	Patch & MTM	1.37 *×* 2.61 *×* 0.65	7	Single	Single (0)	27.2 – 29 (6.4%)	13.7	94
2023^24^	Bow-tie & MTMs	1.65 *×* 2.2 *×* 0.046	1	Single	Double (± 25)	26.3 – 29.8 (9.5%)	12.2	N/A
2024^19^	Dielectric	1.36 *×* 0.58 *×* 0.5	3	Single	Dual (± 19)	26.4 - 27.3 (3.7%)	5.5	> 90
2025^18^	Patch & dipole	1.94 *×* 0.9 *×* 0.07	2	Single	Dual (± 45)	7 - 12.4 (55.9%)	9.7	N/A
2025^20^	Dielectric	1.03 *×* 0.31 *×* 0.5	3	Single	Dual (± 40)	21.2 – 28 (27.6%)	6.3	> 95
This work	Patch	2.3 *×* 1.2 *×* 0.07	1	Dual	Dual (± 40 & ± 24)	24.2 - 30 (22.5%); 32.5 - 43.5 (29%)	9	> 90

$\lambda _{0}$ is the wavelength at the center frequency of the operating band; MBD is main beam direction; Gain is measured; Rad. Effi. is simulated; N/A is data not available in article

For instance, single and double-layered antennas loaded with metamaterials or created slots achieved dual-beam performance and peak gains of more than 5 dBi^16,17,23,24,29,30^. However, they have narrow operating bandwidth.

Multi-layered patch and dielectric antennas improved the gain bandwidth significantly in^18–20,30^. These antennas radiate in endfire or broadside direction and are often loaded with metamaterials in front of printed dipole/bow-tie antennas, resulting in complex geometries. This approach extends the antenna’s size by one wavelength, making it larger compared to the original size.

The proposed antenna demonstrates dual-band dual-beam radiation in the mmWave frequency range using a simple single-layer, via-free dual circular-ring radiator without any metasurfaces. Compared with previously reported mmWave antennas, which typically focus on either single-band dual-beam operation or different radiation characteristics, the proposed design offers a unique combination of dual-band operation, dual-beam radiation, compact size, simple geometry, wide bandwidth, and ease of fabrication. Therefore, when comparing with the most recent reported works such as those in^19,20,24^, the proposed antenna gives an outstanding performance for 5G mmWave applications.

## Conclusion

This paper presents a novel mmWave dual-band and dual-beam antenna for 5G mmWave communications. The main application of this antenna is to compensate for spatial path fading and improve link robustness in MIMO systems. Other potential applications include 5G/6G small-cell base stations, fixed wireless access terminals, automotive radar, intelligent transportation systems, vehicle-to-everything (V2X) communications, and integrated sensing and communications (ISAC). Dual circular-rings fed with a microstrip line achieves dual-beam radiation in the endfire direction (quasi-E plane). Additionally, the paper demonstrates how understanding the surface current distribution can enable redesign of the ground plane to remove unwanted lobes. Moving operation to higher order modes not only reduces size but also results in a wide fractional bandwidth of 21.5% (24.2 to 30 GHz) and 26.3% (32.5 to 44 GHz). At the upper and lower bands, the beams are orientated in the azimuth plane at ± 40$^{\circ }$ and ± 24$^{\circ }$respectively, achieving a peak gain of 9 dBi at 38 GHz. The symmetrical structure and common feeding of the dual circular-rings ensure equal realized gain in both beams, with less than 0.1 dB variation. Removing the need for vias, reducing size and complexity should significantly reduce the cost of manufacture compared to contemporary designs. Furthermore, the simple planar structure makes the antenna suitable for integration into larger phased arrays and beam-steering systems to achieve higher gain and adaptive directional coverage in future wireless networks. In the future, instead of a fixed-beam, adding PIN or Varactor diodes on circular-rings could help to reconfigure the central null between the dual-beams, leading to applications such as interference avoidance.

## Data Availability

Data is provided within the manuscript

## References

[1] Wen, D.-L., Zheng, D.-Z. & Chu, Q.-X. A dual-polarized planar antenna using four folded dipoles and its array for base stations. *IEEE Trans. Antennas Propag.* **64**, 5536–5542. 10.1109/TAP.2016.2623660 (2016).

[2] Lian, R., Wang, Z., Yin, Y., Wu, J. & Song, X. Design of a low-profile dual-polarized stepped slot antenna array for base station. *IEEE Antennas Wirel. Propag. Lett.* **15**, 362–365. 10.1109/LAWP.2015.2446193 (2016).

[3] Alibakhshikenari, M. et al. Dual-polarized highly folded bowtie antenna with slotted self-grounded structure for sub-6 ghz 5g applications. *IEEE Trans. Antennas Propag.* **70**, 3028–3033. 10.1109/TAP.2021.3118784 (2022).

[4] Li, K.-H., Ingram, M. & Rausch, E. Multibeam antennas for indoor wireless communications. *IEEE Trans. Commun.* **50**, 192–194. 10.1109/26.983314 (2002).

[5] Akdeniz, M. R. et al. Millimeter wave channel modeling and cellular capacity evaluation. *IEEE J. Sel. Areas Commun.* **32**, 1164–1179. 10.1109/JSAC.2014.2328154 (2014).

[6] Patel, A., Bansal, A., Panagamuwa, C. & Whittow, W. Wideband end-fire antenna based on modulated grooved surface plasmon polaritons. *Sci. Rep.* **15**, 25144. 10.1038/s41598-025-10667-x (2025). PMC12254507 40646046

[7] Tiwari, R. N. et al. A low-profile sixteen elements four port mimo antenna array for multiband millimeter wave and conformal electronics. *J. Infrared, Millimeter, Terahertz Waves* **46**, 1–28 (2025).

[8] Elabd, R. H., Al-Gburi, A. J. & Megahed, A. A. Compact circular mimo antenna with defected ground structure (dgs) for improved isolation in 5g sub-6ghz mobile systems. *Results Eng.* **27**, 105737 (2025).

[9] Kirthiga, S. & Jayakumar, M. Performance of dualbeam mimo for millimeter wave indoor communication systems. *Wireless Pers. Commun.* **77**, 289–307 (2014).

[10] Wani, Z., Abegaonkar, M. P. & Koul, S. K. Dual-beam antenna for millimeter wave mimo applications. In *2018 IEEE Indian Conference on Antennas and Propogation (InCAP)*, 1–3, 10.1109/INCAP.2018.8770883 (2018).

[11] Khidre, A., Lee, K.-F., Elsherbeni, A. Z. & Yang, F. Wide band dual-beam u-slot microstrip antenna. *IEEE Trans. Antennas Propag.* **61**, 1415–1418. 10.1109/TAP.2012.2228617 (2013).

[12] Chen, H.-Y. & Tao, Y. Performance improvement of a u-slot patch antenna using a dual-band frequency selective surface with modified jerusalem cross elements. *IEEE Trans. Antennas Propag.* **59**, 3482–3486. 10.1109/TAP.2011.2161440 (2011).

[13] Lee, K. F. & Luk, K. M. *Microstrip patch antennas* (World Scientific, 2011).

[14] Li, J.-F., Chen, Z. N., Wu, D.-L., Zhang, G. & Wu, Y.-J. Dual-beam filtering patch antennas for wireless communication application. *IEEE Trans. Antennas Propag.* **66**, 3730–3734. 10.1109/TAP.2018.2835519 (2018).

[15] Dadgarpour, A., Sorkherizi, M. S., Denidni, T. A. & Kishk, A. A. Passive beam switching and dual-beam radiation slot antenna loaded with enz medium and excited through ridge gap waveguide at millimeter-waves. *IEEE Trans. Antennas Propag.* **65**, 92–102. 10.1109/TAP.2016.2631140 (2017).

[16] Tan, J. et al. Design of a dual-beam cavity-backed patch antenna for future fifth generation wireless networks. *IET Microwaves, Antennas Propagat.* **12**, 1700–1703 (2018).

[17] Jiang, H., Si, L.-M., Hu, W. & Lv, X. A symmetrical dual-beam bowtie antenna with gain enhancement using metamaterial for 5g mimo applications. *IEEE Photonics J.* **11**, 1–9. 10.1109/JPHOT.2019.2891003 (2019).

[18] Zheng, W. & Wang, J. A wideband dual-beam patch array antenna with compact size and high efficiency for dual-target wireless communications. *IEEE Antennas Wirel. Propag. Lett.* **24**, 2417–2421. 10.1109/LAWP.2025.3565295 (2025).

[19] Shi, J. et al. Endfire substrate integrated dielectric resonator antenna with dual-beam radiation for millimeter-wave application. *IEEE Antennas Wirel. Propag. Lett.* **23**, 1523–1527. 10.1109/LAWP.2024.3361558 (2024).

[20] Fan, G., Qin, W., Guo, Y., Shi, J. & Jiang, R. Millimeter-wave wideband dual-beam endfire dielectric resonator antenna. *IEEE Antennas Wirel. Propag. Lett.* **24**, 671–675. 10.1109/LAWP.2024.3511659 (2025).

[21] Ding, X.-H., Ji, C., Yang, J.-Y., Yang, W.-W. & Chen, J.-X. A compact dual-polarized endfire dielectric resonator antenna for 5g millimeter-wave terminal. *IEEE Antennas Wirel. Propag. Lett.* **24**, 1288–1292. 10.1109/LAWP.2025.3534655 (2025).

[22] Ni, X.-M. & Ding, X.-H. Compact dual-polarized endfire dielectric resonator antenna for 5g millimeter-wave terminal application. *Progress Electromagn. Res. C* **165**, 199 (2026).

[23] Ullah, H. & Tahir, F. A. A novel snowflake fractal antenna for dual-beam applications in 28 ghz band. *IEEE Access* **8**, 19873–19879. 10.1109/ACCESS.2020.2968619 (2020).

[24] Esmail, B. A. F. & Koziel, S. Design and optimization of metamaterial-based 5g millimeter wave antenna for gain enhancement. *IEEE Trans. Circuits Syst. II Express Briefs* **70**, 3348–3352. 10.1109/TCSII.2023.3263524 (2023).

[25] Das, A., Mathur, S. & Das, S. Radiation characteristics of higher-order modes in microstrip ring antenna. In *IEE Proceedings H (Microwaves, Optics and Antennas)*, vol. 131, 102–106 (IET, 1984).

[26] Handbook, M. A. D. Microstrip antenna design handbook. *R. Garg, P. Bhartia, I. Bahi, and A. Ittpiboon* (2001).

[27] Menzel, W. A new travelling wave antenna in microstrip. In *8th European Microwave Conference* 302–306 (IEEE, 1978).

[28] Oliner, A. A. Leakage from higher modes on microstrip line with application to antennas. *Radio Sci.* **22**, 907–912 (1987).

[29] Jeong, M. et al. Performance improvement of microstrip patch antenna using a novel double-layer concentric rings metaplate for 5g millimeter wave applications. *Int. J. RF Microwave Comput. Aided Eng.* **31**, e22509 (2021).

[30] Wani, Z., Abegaonkar, M. P. & Koul, S. K. High-low-epsilon biaxial anisotropic lens for enhanced gain and aperture efficiency of a linearly polarized antenna. *IEEE Trans. Antennas Propag.* **68**, 8133–8138. 10.1109/TAP.2020.2983787 (2020).

